# Supplementary material for: Modeling glioblastoma heterogeneity as a dynamic network of cell states
Source: Mol Syst Biol. 2021 Sep 16;17(9):e10105. doi: 10.15252/msb.202010105 (PMC8444284; doi:10.15252/msb.202010105)
Supplement: Supplementary file 6 — Source Data for Figure 5 [file MSB-17-e10105-s004.zip › Figure5A_sourcedata/GSEA_3071/hallmarks_stateII.GseaPreranked.1623742536692/HALLMARK_UNFOLDED_PROTEIN_RESPONSE.html]

Details for gene set HALLMARK\_UNFOLDED\_PROTEIN\_RESPONSE[GSEA]

|  || Dataset | state23071 |
| Phenotype | NoPhenotypeAvailable |
| Upregulated in class | na\_pos |
| GeneSet | HALLMARK\_UNFOLDED\_PROTEIN\_RESPONSE |
| Enrichment Score (ES) | 0.31909275 |
| Normalized Enrichment Score (NES) | 1.555682 |
| Nominal p-value | 0.049773756 |
| FDR q-value | 0.05318811 |
| FWER p-Value | 0.44 |
Table: GSEA Results Summary

  

Fig 1: Enrichment plot: HALLMARK\_UNFOLDED\_PROTEIN\_RESPONSE      
 Profile of the Running ES Score & Positions of GeneSet Members on the Rank Ordered List

  

| PROBE | GENE SYMBOL | GENE\_TITLE | RANK IN GENE LIST | RANK METRIC SCORE | RUNNING ES | CORE ENRICHMENT || 1 | CKS1B |  |  | 128 | 0.999 | 0.0209 | Yes |
| 2 | NOP56 |  |  | 250 | 0.824 | 0.0344 | Yes |
| 3 | DKC1 |  |  | 268 | 0.789 | 0.0716 | Yes |
| 4 | EIF2S1 |  |  | 282 | 0.776 | 0.1091 | Yes |
| 5 | MTHFD2 |  |  | 337 | 0.721 | 0.1337 | Yes |
| 6 | EXOSC9 |  |  | 399 | 0.679 | 0.1543 | Yes |
| 7 | EXOSC2 |  |  | 509 | 0.626 | 0.1603 | Yes |
| 8 | DDX10 |  |  | 537 | 0.609 | 0.1856 | Yes |
| 9 | NPM1 |  |  | 544 | 0.605 | 0.2159 | Yes |
| 10 | NOLC1 |  |  | 546 | 0.603 | 0.2473 | Yes |
| 11 | NOP14 |  |  | 693 | 0.534 | 0.2394 | Yes |
| 12 | EXOSC10 |  |  | 747 | 0.517 | 0.2535 | Yes |
| 13 | ERO1A |  |  | 767 | 0.510 | 0.2756 | Yes |
| 14 | SPCS3 |  |  | 792 | 0.501 | 0.2960 | Yes |
| 15 | EIF4EBP1 |  |  | 805 | 0.496 | 0.3191 | Yes |
| 16 | GEMIN4 |  |  | 1005 | 0.443 | 0.2933 | No |
| 17 | SEC11A |  |  | 1019 | 0.438 | 0.3131 | No |
| 18 | EIF4A3 |  |  | 1332 | 0.372 | 0.2558 | No |
| 19 | SLC7A5 |  |  | 1434 | 0.351 | 0.2493 | No |
| 20 | NABP1 |  |  | 1443 | 0.349 | 0.2657 | No |
| 21 | PSAT1 |  |  | 1505 | 0.340 | 0.2685 | No |
| 22 | EXOSC5 |  |  | 1513 | 0.339 | 0.2845 | No |
| 23 | FUS |  |  | 1571 | 0.329 | 0.2878 | No |
| 24 | KHSRP |  |  | 1599 | 0.324 | 0.2981 | No |
| 25 | SHC1 |  |  | 1671 | 0.314 | 0.2971 | No |
| 26 | LSM4 |  |  | 1676 | 0.313 | 0.3126 | No |
| 27 | EIF4A1 |  |  | 1773 | 0.302 | 0.3048 | No |
| 28 | IMP3 |  |  | 1851 | 0.292 | 0.3011 | No |
| 29 | HSPA9 |  |  | 1884 | 0.287 | 0.3084 | No |
| 30 | XBP1 |  |  | 1995 | 0.273 | 0.2956 | No |
| 31 | RRP9 |  |  | 2055 | 0.267 | 0.2950 | No |
| 32 | HYOU1 |  |  | 2367 | -0.266 | 0.2324 | No |
| 33 | DNAJB9 |  |  | 2618 | -0.303 | 0.1867 | No |
| 34 | HSP90B1 |  |  | 2648 | -0.308 | 0.1957 | No |
| 35 | DCP1A |  |  | 2663 | -0.311 | 0.2086 | No |
| 36 | HERPUD1 |  |  | 2777 | -0.331 | 0.1981 | No |
| 37 | SPCS1 |  |  | 2874 | -0.349 | 0.1928 | No |
| 38 | EIF4A2 |  |  | 3289 | -0.453 | 0.1146 | No |
| 39 | HSPA5 |  |  | 3335 | -0.468 | 0.1281 | No |
| 40 | VEGFA |  |  | 3532 | -0.560 | 0.1092 | No |
| 41 | TUBB2A |  |  | 3554 | -0.572 | 0.1341 | No |
Table: GSEA details [plain text format]

  

Fig 2: HALLMARK\_UNFOLDED\_PROTEIN\_RESPONSE: Random ES distribution      
 Gene set null distribution of ES for **HALLMARK\_UNFOLDED\_PROTEIN\_RESPONSE**
